# Supplementary material for: Employing SAE-GRU deep learning for scalable botnet detection in smart city infrastructure
Source: PeerJ Comput Sci. 2025 Apr 30;11:e2869. doi: 10.7717/peerj-cs.2869 (PMC12192925; doi:10.7717/peerj-cs.2869)
Supplement: Supplemental Information 1 [file peerj-cs-11-2869-s001.docx]

| **Acronym** | **Meaning** | **Acronym** | **Meaning** |
| --- | --- | --- | --- |
| IoT | Internet of Things | IDS | Intrusion Detection System |
| DL | Deep Learning | SAE-GRU | Stacked Autoencoder–Gated Recurrent Unit |
| MQTT | Message Queuing Telemetry Transport | CoAP | Constrained Application Protocol |
| LSTM | Long Short-Term Memory | GA | Genetic Algorithms |
| IG | Information Gain | FPR | False Positive Rate |
| FNR | False Negative Rate | TPU | Tensor Processing Units |
| CANFIS | Cascaded Adaptive Neuro-Fuzzy Inference System | MDRL | Modified Deep Reinforcement Learning |
| SVM | Support Vector Machine | DRL | Deep Reinforcement Learning |
| BBO | Billiard Based Optimization | BPEO | Binary Pigeon Optimization |
| ERNN | Elman Recurrent Neural Network | KNN | K-Nearest Neighbor |
| SNN | Sequential Neural Network | OneR | One Rule |
| ZOA | Zebra Optimization Algorithm | DGAN | Dual-channel Graph Attention Network |
| STOA | Sooty Tern Optimization Algorithm | RTSP | Real-Time Streaming Protocol |
| DDoS | Distributed Denial of Service | ReLU | Rectified Linear Unit |
| ROC | Receiver Operating Characteristic | TBPTT | Truncated Backpropagation Through Time |
| TC | Traffic Control | AUC | Area Under the ROC Curve |
| SHAP | SHapley Additive exPlanations | | |

**Table A1**

Glossary of Acronym Notations (Sequenced by Citation Order)
